# Interactions Under Crowding Milieu: Chemical-Induced Denaturation of Myoglobin is Determined by the Extent of Heme Dissociation on Interaction with Crowders

**DOI:** 10.3390/biom10030490

**Published:** 2020-03-23

**Authors:** Khalida Nasreen, Zahoor Ahmad Parray, Shahzaib Ahamad, Faizan Ahmad, Anwar Ahmed, Salman Freeh Alamery, Tajamul Hussain, Md. Imtaiyaz Hassan, Asimul Islam

**Affiliations:** 1 Centre for Interdisciplinary Research in Basic Sciences, Jamia Millia Islamia, Jamia Nagar, New Delhi 110025, India; khalida.mbt@gmail.com (K.N.); zaparray@gmail.com (Z.A.P.); shah.bioinfo@gmail.com (S.A.); faizan.ahmad.jmi@gmail.com (F.A.); mihassan@jmi.ac.in (M.I.H.); 2 Center of Excellence in Biotechnology Research, College of Science, King Saud University, Riyadh-11451, Saudi Arabia; anahmed@ksu.edu.sa (A.A.); salamery@ksu.edu.sa (S.F.A.); thussain@ksu.edu.sa (T.H.); 3 Protein Research Chair, Department of Biochemistry, College of Science, King Saud University, Riyadh-11451, Saudi Arabia

**Keywords:** macromolecular crowding, protein stability, chemical-induced denaturation, myoglobin, binding-induced folding, isothermal titration calorimetry

## Abstract

Generally, in vivo function and structural changes are studied by probing proteins in a dilute solution under in vitro conditions, which is believed to be mimicking proteins in intracellular milieu. Earlier, thermal-induced denaturation of myoglobin, in the milieu of crowder molecule showed destabilization of the metal protein. Destabilization of protein by thermal-induced denaturation involves a large extrapolation, so, the reliability is questionable. This led us to measure the effects of macromolecular crowding on its stability by chemical-induced denaturation of the protein using probes like circular dichroism and absorption spectroscopy in the presence of dextran 70 and ficoll 70 at various pHs (acidic: 6.0, almost neutral: 7.0 and basic: 8.0). Observations showed that the degree of destabilization of myoglobin was greater due to ficoll 70 as compared to that of dextran 70 so it can be understood that the nature of the crowder or the shape of the crowder has an important role towards the stability of proteins. Additionally, the degree of destabilization was observed as pH dependent, however the pH dependence is different for different crowders. Furthermore, isothermal titration calorimetry and molecular docking studies confirmed that both the crowders (ficoll and dextran) bind to heme moiety of myoglobin and a single binding site was observed for each.

## 1. Introduction

Generally, it is assumed that the folding methods and biophysical and structural properties of an isolated protein monitored in dilute solutions (in vitro) are similar to the cellular conditions (in vivo). However, there are two major differences between in vitro and in vivo protein folding. First, there is a complete machinery available in the cell for proper folding of the protein like chaperones, etc. In the case of small proteins like cytochrome *c* (cyt*c*), myoglobin (Mb), lysozyme and ribonuclease A, chaperones are not required and the folding of protein is reversible and quick, even in absence of such folding machinery. The second and important difference is the presence of the highly crowded intracellular environment, due to the huge quantity of insoluble and soluble biomolecules, which comprises of proteins, nucleic acid, carbohydrates, osmolytes, ribosomes, and the architecture of a cell [1].

Crowding can have significant effect on structure, stability, functional activity, and aggregation of proteins; it may affect kinetics of protein folding, protein–protein interactions, and other complex physiological processes undergoing in a cell [2]. Since the intracellular environment is highly crowded, it becomes very imperative and interesting to know how protein folds and functions in the cellular environment. Various natural and artificial crowder molecules are used with the purpose to investigate the structural, functional changes and type of interactions induced by crowding, in order to mimic cell like conditions [2,3,4]. As the cellular environment is composed of various biomolecules, which differ in shape, size and chemical nature [5], crowders having different shape, size and nature were used in order to study the effect of crowding [6,7]. The dependence of crowding effect on the concentration, size, shape and nature of crowding agents are very important attributes which must be considered while studying protein folding [8].

Many findings are there to demonstrate the crowding effect on the conformations of proteins [9,10,11,12,13,14,15,16,17]. In some cases, crowding agents have significantly altered the structural contents of several proteins while the conformational properties of some proteins have remained unaffected in the presence of crowding environment. The secondary structural contents of *Desulfovibrio desulfuricans* flavodoxin [11], apoflavodoxin [10], bovine pancreatic RNase A [18], holo α-lactalbumin from bovine milk [18], rabbit muscle creatine kinase [14] and *Borrelia burgdorferi* VIsE [13] in their native state, increased due to ficoll 70 and/or dextran 70 and is concentration dependent, however the thermally and chemically denatured state remained unaffected. In contrast, the secondary structure of the monomeric multi-copper oxidase Fet3p from *Saccharomyces cerevisiae* [19] and lysozyme from hen egg white [18] were not affected by ficoll 70 [18,19] and dextran 70 [20], respectively. Conversely, the reports had shown that there was decrease in the secondary structure of rabbit muscle creatine kinase due to dextran 70 [21]. In addition, it has been demonstrated that dextran 20 shows unique character in the unfolded or partially folded proteins including variant of *Streptococcus magnus* immunoglobulin G binding domain of protein L, [16], *S. Cerevisiae* Fet3p [19] and F10C/W74F small ribosomal variant protein (S16) [15], leading to an increase in their secondary structures. Although dextran 20 was unable to show such a change in the secondary structure of apoflavodoxin from *Azotobacter vinelandii* [12] and ubiquitin, significant changes were observed in its denatured state [22]. 

Most of the time, the phenomenon of macromolecular crowding increases the thermodynamic stability of proteins [9,10,11,12,13,14,15,16,17,18,20,23,24,25,26,27,28,29,30,31,32]. However, the recent study of Ashima Malik and collaborators [33] observed that effects of the crowding agents on Mb leads to deviation from the general belief that crowding agents (synthetic) are always stabilizing in nature. They did thermo-chemical denaturation experiments using urea (different concentrations) in the presence of crowders. They showed that ficoll 70 particularly destabilizes Mb. Both chemical- and heat-induced denaturation revealed different effects on the unfolded ensemble of Mb in presence of the crowders. The concentration of 200 mg/mL of the crowders was observed using heat-induced denaturation method, to have destabilizing effect on Mb. The data acquired from the heat-induced chemical denaturation curves involves a long data extrapolation measured in the range of temperature above from 25 °C, and this range of extrapolation increases whilst co-solutes are taken. Thus, the Δ*G*_D_^0^ value obtained may show some error, which shows deviation in *T*_m_ (the midpoint of thermal denaturation) in the presence of co-solutes [34]. In this work, we have performed experiments at 25 °C at different pH values using different concentrations of ficoll 70 and dextran 70 on Mb, using a more accurate method, i.e., under chemical (GdmCl and urea)-induced denaturation.

In this study, the effect of ficoll 70 and dextran 70 was observed on the structure and stability of Mb at variable pH values (6.0, 7.0 and 8.0) under chemical-induced denaturing conditions (GdmCl and urea) using specific probes of absorption and circular dichroism (CD) spectroscopy. Furthermore, interaction studies of ficoll 70 and dextran 70 with Mb was investigated using isothermal titration calorimetry (ITC) and molecular docking studies.

## 2. Materials and Methods

### 2.1. Materials

Lyophilized myoglobin (horse heart), ficoll 70, dextran 70 and sodium salt of cacodylate were procured from Sigma Aldrich Private Ltd., (St. Louis, MO, USA). Potassium ferricyanide, ethylene diaminetetraacetic acid (EDTA), potassium chloride (KCl), hydrochloric acid (HCl) and sodium hydroxide (NaOH) pellets were bought from Merck (India). Guanidinium chloride (GdmCl) and ultrapure urea were procured from ICN Biomedical Inc., Irvine, California; United States of America (USA). Filters (0.22 µm pore size) were procured from Millipore Corporation and parafilm from American National Co., Chicago, IL, (USA). 

### 2.2. Methods

#### 2.2.1. Preparation of Protein Solutions

Stock solution of Mb (5 mg/mL) was prepared in 0.1 M KCl. The solution was then oxidized using 0.01% potassium ferricyanide (K_3_Fe(CN)_6_) [35]. In order to remove excess of potassium ferricyanide and additional salts present in the protein (lyophilized form), the solution prepared was dialyzed comprehensively against changes of 0.1 M KCl in conditions of pH 7.0 and 4 °C. Solution of protein was then filtered using 0.22 µm Millipore filter paper. Concentration of Mb was determined using molar absorption coefficient value of 171,000 M^−1^cm^−1^at wavelength 409 nm [36], using Beer–Lambert’s law:

*A* = *εcl*, (1)

(conc. of protein, *c* = *A*/*εl*) where ε is molar absorption coefficient of protein (M^−1^cm^−1^) and l is path length of cuvette).

#### 2.2.2. Preparation of Working Solution of the Protein 

A quantity of 3–4 µM working concentration was prepared for absorption spectroscopy measurements from stock concentration using N1V1 = N2V2. The samples were measured in triplicate so the samples of each single spectrum were observed at 3 values from which average value was plotted. The mean error was calculated by the difference in the average value to the three observed values of triplicate samples. The error values were used to get the average value to be final error.

#### 2.2.3. Denaturant Stock Solution Preparations

The required amount of denaturants (GdmCl and urea), were dissolved in the desirable buffers used in the experiments (0.1 M KCl and 0.05 M cacodylic acid). The pHs of these solutions were then adjusted using NaOH or HCl as per used in the experiments. The solutions were then filtered through Whatman filter paper no. 1. The Abbe refractrometer was then used to check refractive index, and plotted using the tabulated values of the refractive index reported for GdmCl by Nozaki [37] and for urea by Pace [38]. GdmCl stock solution was stored for further use while urea stock solution was always prepared fresh because urea tends to decompose forming cyanate and ammonia ion [39], reacts with sulphydryl groups and ammonia of the protein leads irreversible inactivation [40]. 

#### 2.2.4. Preparation of Crowder Stock Solution

Concentrated stock solution of crowders (ficoll and dextran) was prepared by dissolving required amount of crowder in distilled water. KCl and cacodylic acid were added to the solution in order to get a final concentration of 0.1 M and 0.05 M respectively. The NaOH or HCl were used to equilibrate pH of the solutions as required in the work. In order to remove the impurities, the solutions were filtered using Whattman filter paper and then stored at 4 °C. The concentration of the crowders, i.e.,ficoll 70 and dextran 70, were calculated using the reported refractive increment value [41,42]. For experiment measurements, each solution sample was thoroughly mixed and incubated overnight at room temperature which is an optimum time required to have changes in the protein in the presence of the additives used. We would like to mention that we have observed that incubating samples for 6 h or/and overnight provide similar results. Even one-week incubation gives the similar result. In this case, all the samples were incubated overnight.

#### 2.2.5. Spectral Measurements and Analysis of Isothermal Transition Curves

##### Absorption Spectroscopy

Spectral measurements were carried out in Jasco-660 ultraviolet (UV)/Visible spectrophotometer equipped with Peltier type temperature controller (ETCS-761), using cuvette of 1 cm path length. Temperature of the cell was set at ±25 °C by thermo-stated circulating water bath. The quantity of 3–4 µM of the protein concentration was used for the spectral measurements, which were carried in the wavelength range of 450–350 nm. Correction of baseline was carried out continuously using the working buffer. The spectrum of the native protein was subtracted from that of the denatured protein in order to get the difference spectrum. Wavelength at which maximum change was observed in the difference spectrum was further used to monitor the denaturation curves, i.e., 409 nm. The raw data was converted into molar absorption coefficient, *ε* using Equation (1).

The absorbance data were changed to molar absorption coefficient at each wavelength (nm) to make it concentration independent. The change in the absorption coefficient (Δ*ε*_409_) was used as probe to plot denaturation curves at all concentrations to make the measurements concentration independent.

##### Far-UV Circular Dichroism Spectroscopy 

Circular dichroism studies were made using Jasco J-1500 CD spectropolarimeter (JASCO International Co. Ltd, Tokyo, Japan) equipped with a Peltier type temperature controller attached to circulating water bath (MCB-100),using a cell of path length 0.1 cm. Protein concentration used for CD measurements was 6–7 µM. The equipment was regularly calibrated using D-10 camphorsulphonic acid. Baseline correction was done using buffer. Then, 3–5 accumulations of each sample including the baseline were taken in order to improve the CD signals, which is the average of each spectrum. Nitrogen gas was continuously flushed at the rate of 3–5 lit/min inside the cuvette chamber in order to minimize the noise level. Far-UV CD spectra were scanned in the range of 250–200 nm. The raw data from CD (milli degree) were changed into mean residual ellipticity completely using the relation [43]:

[*θ*]_λ_ = *M*_0_*θ*_λ_/10*lc*, (2)

where *θ*_λ_ is the ellipticity observed in millidegrees at wavelength of λ, *l* is the path length of the cuvette in centimeters, *M*_0_ and *c* are the mean residue weight and the concentration of the protein respectively. The ellipticity at 222 nm, [*θ*]_222_ was used as probe to plot denaturation curves at all concentrations to monitor secondary structure in order to make the measurements concentration independent.

Reversibility of denatured Mb was checked by measuring optical property using high concentration of denaturant, and then dialyzing it with respective buffer.

##### Analysis of Isothermal Denaturation Curves

Analysis of isothermal denaturation curves was done in order to find out the stability of protein in the absence and presence of co-solute. Protein stability is defined as the change in Gibb’s free energy when an unfolded polypeptide chain folds into its stable native conformation. 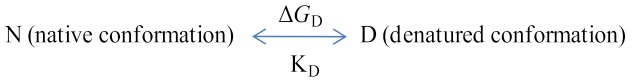

Δ*G* = *G*_D_ − *G*_N,_ (3)

*K*_D_ = [D]/[N]. (4)

Analysis of isothermal transition curves was based on two assumptions: (1) the isothermal denaturation process is reversible, and (2) the denaturation proceeds through two-state mechanism, having equilibrium between N (native conformation) ↔ D (denatured conformation).

The change in Gibb’s free energy (∆G_D_) for folding-unfolding was analyzed using the relation:

∆*G*_D_ = −*RT* ln *K*_D,_ (5)

where, *R* is the gas constant (1.98 cal deg^−1^ mol^−1^) and *T* is the absolute temperature in Kelvin (*K*) and *K*_D,_ dissociation constant, can be computed using the relation:

*K*_D_ = *f*_D/_(1 − *f*_D_) = (*y* − *y*_N_)/(*y*_D_ − *y*) (6)

where, *y* is the optical property measured at the specific concentration of denaturant, *y*_N_ and y_D_denotes the native and denatured states respectively. These are points in the transition region, obtained by linear extrapolation of the pre- and post-transition region and attained under the same experimental conditions where *y* has been observed.

$\Delta G{{}^{\circ}}_{N↔D}$ was plotted against the molar concentration of each denaturant and Δ*G*_D_° was estimated from the least square analysis using the relation:

∆*G*_D_ = ∆*G*_D_° − *m*[d], (7)

where, $ƊG{{}^{\circ}}_{N↔D}$ is the value of ∆*G*_D_ in the absence of denaturant and *m* is the slope of the line i.e., (∂Δ*G*_D_/∂[d])_T, P_ and [d], the molar concentration of denaturant. The transition curve mid-point, *C*_m_ was analyzed from *C*_m_ = ∆*G*_D_°/*m*.

Alternatively, the entire equilibrium transition curve was fitted and analyzed to a two-state unfolding model in order to get the values of Δ*G*_D_°, *m* and *C*_m_ using the relation given by Santoro and Bolen:

(8) $$y\left(d\right)=\frac{y_{N}\left(d\right)+y_{D}\left(d\right)*\;\mathrm{Exp}\left[-\left(\Delta G_{D}{}^{\circ}+\;m\;\left[d\right]\right)/\mathrm{RT}\right]}{1+\;\mathrm{Exp}\;\left[-\left(\Delta G_{D}{}^{\circ}+\;m\;\left[d\right]\right)/\mathrm{RT}\right]}$$

where, *y*(d) is the optical property observed at a specific concentration of denaturant, *y*_N_ and *y*_D_ are the native and denatured state optical properties respectively in similar experimental conditions where *y*(d) was measured. Δ*G*_D_° is change in Gibb’s free energy of native protein, *m* is the slope of ∂Δ*G*_D_ versus ∂[d] plot, *T* is the temperature in Kelvin and *R* is the universal gas constant.

#### 2.2.6. Isothermal Titration Calorimetry 

Isothermal titration calorimetry (ITC) experiments were performed in MicroCal VP-ITC system at pH 7.0 and 25 °C. The concentration of dextran 70 and Mb were taken in the ratio of 1:10 respectively. 2 mL of crowder solution (200 µM) was taken inside the sample cell and in injection syringe 280 µL of protein solution (20 µM) was loaded. Stirring speed of the syringe was 372 rpm. There was negligible heat of dilution between buffer and the crowders and baseline was subtracted from the raw data (protein and crowders) to get final results. MicroCal ORIGIN software, version 7.0 (Malvern Panalytical, (Malvern, UK) was used in order to analyze the data and find out the binding model that could be best fitted to the data. Raw data were processed using Origin version 7.0 software and was fitted by oneSite model using Microcal.

#### 2.2.7. Computational Methods

In silicoassessment of binding between ficoll or dextran with Mb (PDB ID: 1ymb) was done by means of AutoDock4, software for docking and maestro visualizer (Schrodinger-version 10.6, (SCHRODINGER, New York, NY, USA), 2D interaction plot [44]. The preparation of input file was done by chemdraw and MM2 force field (ChemBio3D and Chemdraw version 12, Perkin Elmer, Akron, OH, USA) was taken for minimization of energy. The conversion of .pdb was done into .pdbqt (receptor and ligand file). Autogrid 4 module was used to cover all of the amino acid residues of the protein. The grid dimensions X, Y, Z were set at 90X, 90Y and 90Z Å (receptor axes coordinates), and was allocated the grid space size as 0.375 Å. Lamarckian Genetic Algorithm (LGA) was used for docking simulations in order to turn out the finest conformation of the receptor and the ligand. The docked pose was visualized using PyMOL vo.99 (Delano Scientific LLC 400 Oyster Point, South San Francisco, CA, USA).

## 3. Results

Primarily, so many studies have been focused on unfolding holo-Mb under different conditions (pH, temperature, denaturants like urea and GdmCl) because of large absorbance changes go with loss of heme and denaturation. Though, result interpretations are indistinct as in most cases heme loss resistance than the apo-protein structure stability is being considered. The concentration of a denaturant is systematically varied in the equilibrium experiments, the concentrations and properties of folded–unfolded and intermediate conformations at equilibrium are observed [45].

### 3.1. GdmCl- and Urea-Induced Mb Denaturation in the Absence and Presence of Ficoll 70 at Different pH Values

At constant temperature, Soret absorption spectra (450–350 nm) of myoglobin in crowded environment at various pH values (pH 6.0, 7.0 and 8.0), in the absence and presence of various concentrations of GdmCl and urea were measured. The crowded environment was established by providing different concentrations of ficoll 70 or dextran 70. In myoglobin, porphyrin ring of the heme moiety acts as a chromophore and shows characteristic absorption peak at 409 nm, which originates from the interaction between heme and globin (folded protein) and is a signature of the native myoglobin [1]. Absorption of Mb in the Soret region is extremely sensitive to the milieu of heme and therefore generally has been exploited to monitor disruption in the heme pocket due to change in experimental condition [2,3]. GdmCl-induced denaturation of Mb in the presence of ficoll 70 (Figure S1A) and dextran 70 (Figure S1B) was measured in the wavelength range 350–450 nm. Figure S1 clearly shows that heme is getting perturbed in the presence of crowders at pH 7.0. We observed similar trend at pHs 6.0 and 8.0 (data not shown).

GdmCl- and urea-induced denaturation of Mb in the presence of ficoll 70 was monitored by observing changes in *ε*_409_ at different pH values. It has been observed that denaturation of Mb is reversible under these conditions; *Y*_N_ (171,000 m^−1^ cm^−1^) depends on neither pH nor GdmCl and ficoll 70 concentrations; and *Y*_D_ measured at different pH values is independent of [GdmCl] in presence of ficoll 70 at all concentrations but shows a slight dependence on pH. Values of Δ*ɛ* at 409 nm and mean residual ellipticity at 222 nm were plotted as a function of denaturant concentration in order to obtain the transition curves.

#### 3.1.1. Absorption Measurements

**Figure 1:**
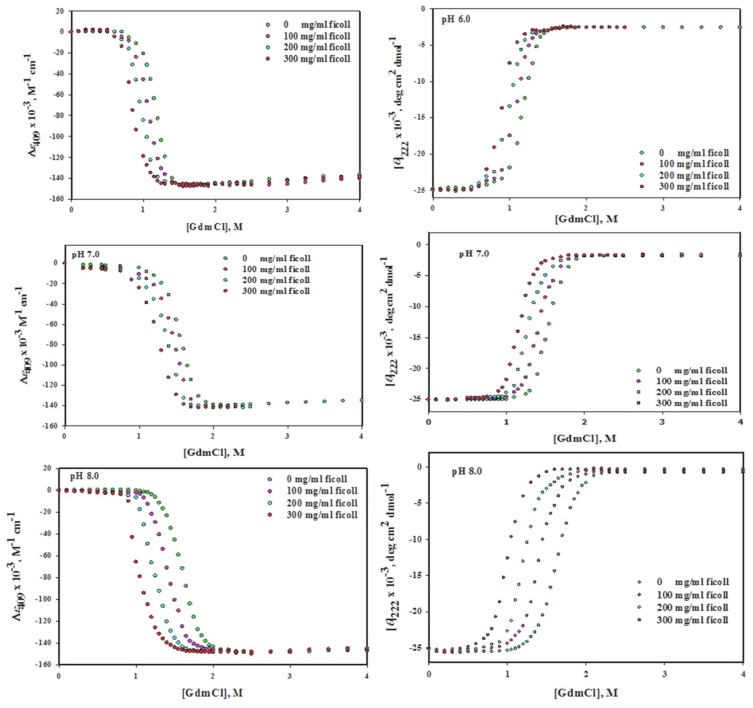
GdmCl-induced denaturation of myoglobin in the absence and presence of different concentrations of ficoll 70 at different pH values.

**Figure 2:**
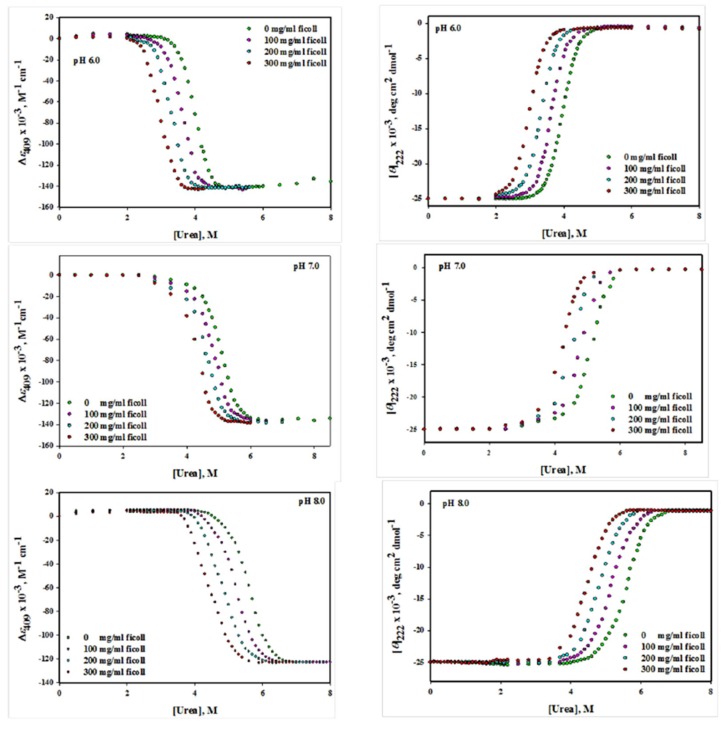
Urea-induced denaturation of myoglobin in the absence and presence of different concentrations of ficoll 70 at different pH values.

The Figure 1 (left panel) shows the changes of (*ε*)_409_ values at different pH values (6.0, 7.0 and 8.0) upon GdmCl-induced denaturation in the absence and presence of various concentrations (0, 100, 200 and 300 mg/mL) of ficoll 70. Figure 2(left panel) shows the urea-induced denaturation of Mb in the absence and presence of ficoll 70 that has been monitored by using the absorption coefficient value at 409 nm, *ε*_409_. It can be seen from the above figures that the pre- and post-transition region are well defined. The protein unfolding was observed to be reversible at all concentration of denaturants in the absence and presence of ficoll 70. The entire data of each denaturation curve was explored for Δ*G*_D_°, *m* and *C*_m_ by means of non-linear least square method (Equation (8)). Value of Δ*G*_D_° and *C*_m_ of Mb in the native state and in the presence of ficoll 70 were measured and is given in parenthesis in Table 1.

#### 3.1.2. Circular Dichroism Measurements

The Figure 1(right panel) shows the changes in [*θ*]_222_ values at different pH values (6.0, 7.0 and 8.0) upon GdmCl-induced denaturation with and without ficoll 70 (0, 100, 200 and 300 mg/mL) while Figure 2(right panel) shows the urea-induced denaturation of Mb with and without ficoll 70, which has been monitored by following the value of mean residual ellipticity at 222 nm. It can be seen that well-defined pre-transition and post-transition regions do exist in the figure. The protein was reversible at all concentration of denaturants in the absence and presence of ficoll 70. The Δ*G*_D_°, *m* and *C*_m_ were analyzed from entire data of each denaturation curve using non-linear least square method (Equation (8)), Δ*G*_D_° and *C*_m_ of Mb in the absence and presence of ficoll 70 were measured and are provided in Table 1.

### 3.2. GdmCl-Induced and Urea-Induced Denaturation of Mb in the Absence and Presence of Dextran 70 at Different pH Values

#### 3.2.1. Absorption Measurements

**Figure 3:**
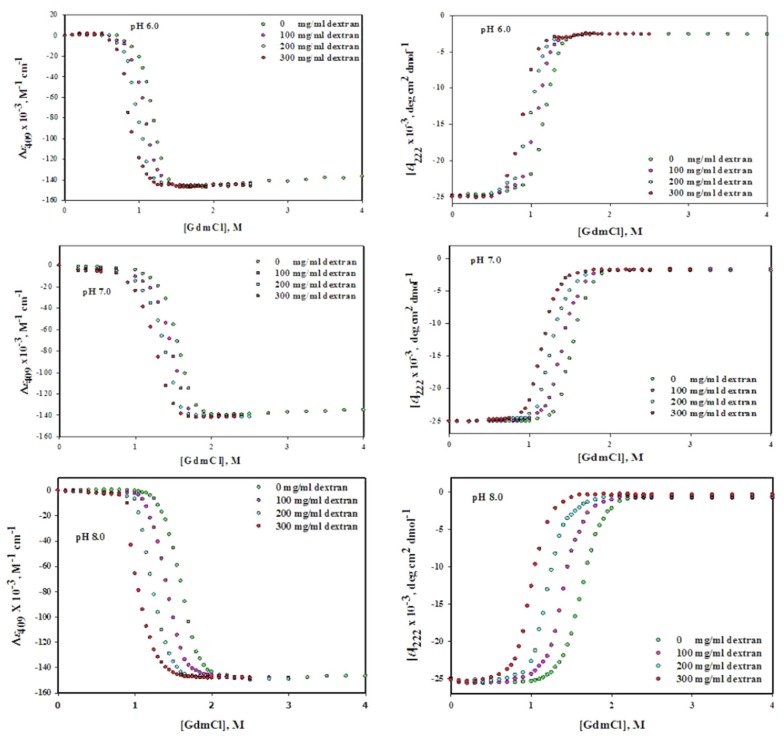
GdmCl-induced denaturation of myoglobin in the absence and presence of different concentrations of dextran 70 at different pH values.

**Figure 4:**
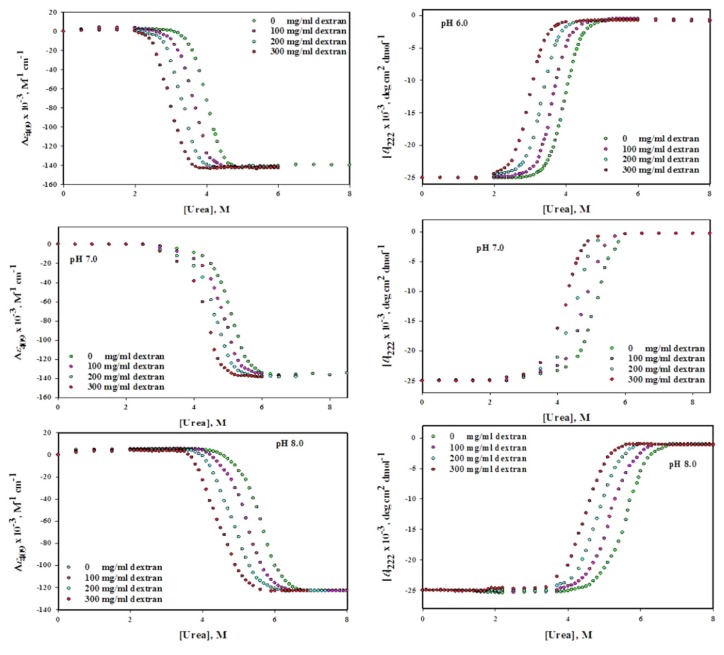
Urea-induced denaturation of myoglobin in the absence and presence of different concentrations of dextran 70 at different pH values.

The Figure 3 (left panel) shows GdmCl-induced denaturation of Mb at pH values 6.0, 7.0 and 8.0 in the absence and presence of different concentrations of dextran 70 (0, 100, 200 and 300 mg/mL). This figure shows the changes in (*ε*)_409_ values against each concentration of GdmCl in absence and presence of dextran 70. Figure 4(left panel) shows the changes in (*ε*)_409_ of Mb due to urea-induced denaturation in the presence dextran 70 at different concentrations (0–300 mg/mL) under various pH conditions (6.0, 7.0 and 8.0). It can be seen that well-defined pre-transition and post-transition regions do exist in this figure. The unfolding of proteins was found to be reversible at all concentration of denaturants in the absence and presence of dextran 70. The Δ*G*_D_°, *m* and *C*_m_ were analyzed from the entire data of each denaturation curve using non-linear least square method (Equation (8)). Value of Δ*G*_D_° and *C*_m_ of GdmCl and urea denatured Mb in the presence of different concentrations of dextran 70 (0–300 mg mL^−1^) at different pH values are given in Table 2.

#### 3.2.2. Circular Dichroism Measurements

The Figure 3 (right panel) shows GdmCl-induced denaturation of Mb at pH values of 6.0, 7.0 and 8.0, in the absence and presence of different concentrations of dextran 70 (0, 100,200 and 300 mg/mL). This figure shows changes in [*θ*] _222_ values against each concentration of GdmCl in absence and presence of dextran 70. Figure 4 (right panel) shows the urea-induced denaturation of Mb at pH values (6.0, 7.0 and 8.0), in the absence and presence of dextran 70 monitoring the mean residual ellipticity at 222 nm. The pre-transition and post-transition regions in the figure are well defined. The protein was found to be reversible at all denaturant concentrations in the absence and presence of dextran70. Δ*G*_D_°, m and *C*_m_ were analyzed from denaturation curves using non-linear least square method (Equation (8)). Value of Δ*G*_D_° and *C*_m_ of GdmCl- and urea-induced denatured Mb in the presence of different concentrations of dextran 70 (0–300 mg ml^−1^) at different pH values given in parenthesis in Table 2.

### 3.3. Interaction Studies

#### 3.3.1. Isothermal Titration Calorimetry Measurements

**Figure 5:**
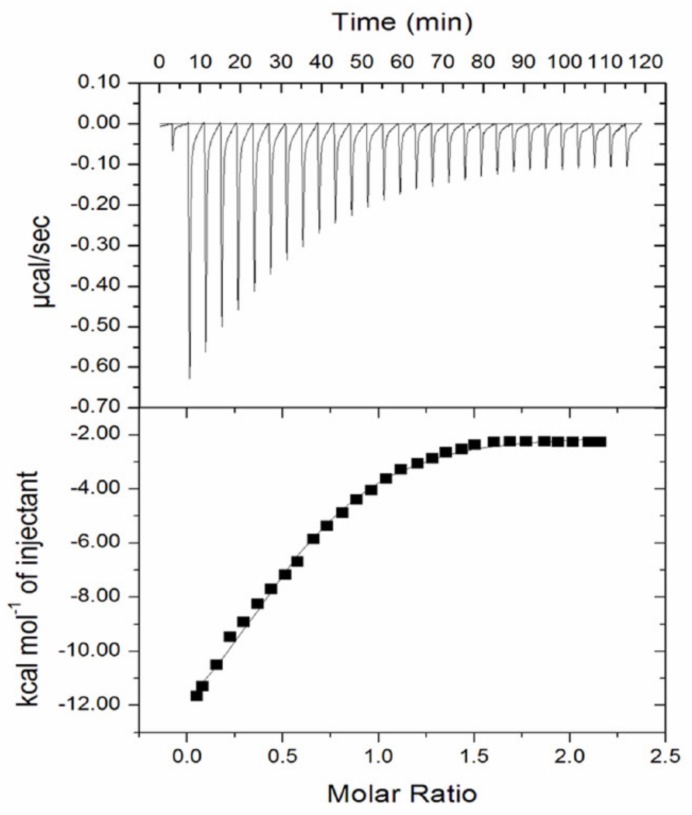
Isothermal titration calorimetry profile for titration of dextran 70 into myoglobin.

Earlier, we have reported that ficoll 70 binds with Mb [46]. To know whether dextran 70 also binds to Mb; and to delineate the chemical basis of structural changes in Mb due to dextran 70, isothermal titration calorimetry (ITC) was carried out to find out possible binding between Mb and dextran 70. Mb in the syringe was titrated inside the cell having dextran 70 (Figure 5). The upper panel of this figure shows power versus time after injection of Mb in cell having dextran 70. The figure shows power per mole of the injectant (kcal mol^−1^) versus its molar ratio at lower panel side. During titrations of Mb into dextran 70 the initial injections produces large negative enthalpic change that reduced upon further addition of Mb into dextran 70. The binding model, which could fit to the data, was “oneSite” binding model. This binding model was used in order to find out thermodynamic parameter, i.e., the association constant (*K*_a_), binding enthalpy (Δ*H*) and stoichiometry (N). The values of the association constant (*K*_a_),change in entropy (Δ*S*), Gibb’s free energy change (Δ*G*), binding enthalpy (Δ*H*) and stoichiometry (N) for dextran 70 binding to Mb are given in Table 3. This binding model presumes that one or additional ligand molecule binds with equivalent affinities to a single site. The result from ITC suggests that interaction occurs between dextran 70 and Mb.

#### 3.3.2. Molecular Docking Studies

From the ITC studies, we could demonstrate that interaction occurred between the ligand and macromolecule; and measured the thermodynamic parameters associated with binding. However, ITC could not give much information about the mechanism of interaction. To know the basis of interaction and the site where the ligand binds to macromolecule (Mb), molecular docking studies were performed. There are certain limitations while docking dextran 70. Dextran 70 is a large rod-shaped molecule while during docking the structure was showing as linear chain. So, we took a dimer of dextran to understand the mechanism of interaction between the protein and the ligand.

The interaction between dextran (dimer) and Mb was studied by molecular docking using AutoDock4 software. *In silico* study shows that interaction was significant between myoglobin and dextran dimer as can be seen in Figure S2. Figure S2A shows the surface view and Figure S2B shows protein (in cartoon model), where heme and amino acid residues of the protein interacts with dextran dimer (in ball and socket model). The computational studies Figure S3 shows the 2D plot of Mb docked with dextran dimer produced by maestro (Schrodinger), which depicts metal coordination, bonding, charge, hydrophobicity, polarity, etc., on the protein in presence of the ligand. This figure revealed that dextran dimer interacts with the heme of Mb via 3 H-bonds with bond distances of 2.1, 1.8 and 1.9 Å, Lys 63 with bond distances of 3.0 and 2.7 Å and Lys 45 with bond distances 2.6 and 2.9 Å. Binding free energy released on interaction of Mb and dextran dimer on docking was calculated to be −2.5 kcal/mol, indicates favorable interactions between Mb and dextran dimer. The type of interaction between ficoll 70 and Mb was already reported where molecular docking was performed using AutoDock4 software [46]. *In silico* studies had showed that notable interaction existed between Mb and ficoll 70 [46].

## 4. Discussions

Thermal- and chemical-induced denaturation of myoglobin is determined by the degree of dissociation of its prosthetic group “heme”, which is pursued right away through rapid unfolding of the globule at the high temperatures and concentration of denaturants [47,48]. The resistance of holo-Mb to unfolding depends on intrinsic stability of tertiary structure of the apo-protein and interaction strength of globule with heme. Reason is the affinity of apoMb for heme is enormous (≈3 × 10^14^ M^−1^), that is so, the holo-protein is more firm to denature than the apo-protein [49,50,51].

The thermodynamic investigation of protein denaturation is one of the functions for valuation of the stabilization free energy of proteins. It has been defined as the free energy entailed for converting the protein from its native three-dimensional structure to the completely denatured state. The latter state has been observed in concentrated solution of GdmC1, guanidinium thiocyanate (GdmCNS), and urea [52].

Macromolecular crowding has shown great impact on the structure of protein, protein’s function, protein stability, protein folding, binding of ligand to protein, protein–protein interaction, molten globule formation and protein aggregation [2,7,9,46,53,54,55,56,57,58]. Here, the chemical denaturation of Mb was observed under crowding conditions i.e., ficoll 70 and dextran 70, using denaturing agents (GdmCl and urea) at pH values of 6.0, 7.0 and 8.0 at 25 °C. Analysis of isothermal transition curves was based on two assumptions: (1) the isothermal denaturation process is reversible, and (2) the denaturation process proceeds through two-state mechanism, having equilibrium between:

**N** (native conformation) ↔ **D** (denatured conformation).

Reversible process means that the process of unfolding (from **N** state to **D** state) and refolding (from **D** state to **N** state) of protein follows the same path. Reversibility of Mb was checked in the absence and presence of each crowder and it was reversible in each case. We have also validated the two state unfolding assumptions of holo-Mb using spectroscopic techniques (absorption spectroscopy and circular dichroism) in the absence and presence of each crowder. Table 1; Table 2 show the thermodynamic parameters acquired from GdmCl- and urea-induced denaturation in the presence of each crowder at different concentration (0–300 mg mL^−1^) using two different probes at different pH values (Figure 1, Figure 2, Figure 3 and Figure 4). It was observed that the thermodynamic parameters obtained from set of three experiments and from different probes (CD and absorbance) were almost identical and were within the limit of experimental error. This indicates that the process of denaturation proceeds through two-state mechanism, having equilibrium between **N** (native conformation) and **D** (denatured conformation). In this study, reversibility and two-state unfolding mechanism [59,60] is reliable in the absence and presence of crowders. The study of Rahman et al. [61,62] and Anjum et al. [63,64] from the same laboratory also reports that the unfolding of Mb at different pH values is a two state reversible process. In the light of earlier finding and this investigation, we conclude that unfolding of Mb is reversible two state processes both in the absence and presence of each crowder at different pH values.

Since the Mb structure is disrupted in the presence of ficoll 70, we wanted to test if the structural effects studied on the native conformation correlate with the Mb stability in the presence of ficoll 70. Although there was no significant effect of dextran 70 on the structure of Mb we wanted to know whether it has any effect on the stability of Mb or not. In this study, we have monitored the effects of various attributes of macromolecular crowding agents such as effect of shape of crowding agent and nature of the crowding agent on the structure and stability of protein as well as effect of various concentration of crowding agent on the structure and stability of protein. Thus GdmCl- and urea-induced denaturation of Mb was done in variable concentration of ficoll 70 and dextran 70 (0–300 mg ml^−1^) at three pH values (6.0, 7.0 and 8.0) and at 25 °C. The midpoint of chemical denaturation, *C*_m_ and the change in Gibb’s free energy Δ*G*_D_° of a chemical induced denaturation curve for a protein are said to be the most frequently used as protein stability indicator. It was observed from the chemical denaturation curve of Mb (both urea-induced and GdmCl-induced denaturation) that shift in the transition occurs towards lower denaturant concentration as the concentration of each crowder was increased (Figure 1, Figure 2, Figure 3 and Figure 4; Table 1, Table 2 and Table 3). This observation implies that the midpoint of chemical denaturation of Mb decreases with increase in the concentration of each crowding agent. Our aim was to investigate the effect of variable concentrations of each crowder molecule on the values of Δ*G*_D_° and *C*_m_, which are the key thermodynamic parameters signifying protein stability. The values of Δ*G*_D_° and *C*_m_ of Mb decrease as the concentration of each crowder molecule was increased. The decrease in the value of Δ*G*_D_° and *C*_m_ in the presence of each crowder signifies destabilization of Mb.

Table 4 displays the comparison of thermodynamic stability parameters (ΔΔ*G*_D_°) of highest concentration of ficoll 70 and dextran 70 effects on Mb. Actually, the value of Δ*G*_D_° decreases from 11.10 kcal mol^−1^ to 9.08 kcal mol^−1^ upon increasing ficoll 70 concentration from 0 to 300 mg/mL a pH 7.0. In case of dextran 70, the value of Δ*G*_D_° decreases from 11.10 kcal mol^−1^ to 9.68 kcal mol^−1^ upon increasing the concentration from 0 to 300 mg/mL at pH 7.0.The results at pH 6.0 showed that ficoll 70 (300 mg/mL) decreases the value of Δ*G*_D_° from 10.13 to 8.78 kcal mol^−1^. In case of dextran 70 the value of Δ*G*_D_° decreases from 10.13 kcal mol^−1^ to 9.05 kcal mol^−1^ upon increasing its concentration from 0 to 300 mg/mL at pH 6.0. Similarly, at pH 8.0 it was observed that ficoll 70 at concentration of 300 mg/mL decreased the value of Δ*G*_D_° from 10.23 to 8.81 kcal mol^−1^. In case of dextran 70, the value of Δ*G*_D_° decreases from 10.23 to 9.03 kcal mol^−1^ upon increasing its concentration from 0 to 300 mg/mL at pH 8.0. From these observations, it can be suggested that the degree of destabilization was more due to ficoll 70 contrast to that of dextran 70. It is assumed that dextran 70 behaves as rod like structure while ficoll 70 has a spherical structure [65]. It has been reported that dextran 70 owing to its rod like structure excludes greater volume than that of ficoll 70 [66]. However, the difference in their structure could not be the reason behind their different degree of destabilization as instead of volume exclusion, here soft interactions are playing a major role. We may hypothesize that ficoll 70 disrupts the tertiary structure of the protein which lead to greater destabilization than that of dextran 70. Further, there is a clear trend in ΔΔ*G*_D_° with respect to both the crowders in these experimental results. It was observed that the degree of destabilization was more due to ficoll 70 than that of dextran 70.

Ficoll 70 is a branched heterogeneous polymer of epichlorohydrin and sucrose while dextran 70 is a branched homogeneous polymer of glucose. Since in the case of soft interactions, chemical interactions are happening, it may be suggested that the natures of crowding agents can be a significant factor in the degree of destabilization of proteins. Crowding agents, i.e., low molecular size PEG 400 Da and intermediate sizes (PEG 10 kDa, PEG 8 kDa) [3,57,58], ficoll, dextran [33] and proteins (lysozyme and BSA) [3] also destabilize Mb, and in another report it shows that glucose, an osmolyte, also destabilizes Mb [67]. However, it was reported that glucose and sucrose, the monomeric constituents of dextran- and ficoll-based crowder prevents the heme dissociation at higher concentrations [3]. Thus, the Crowder’s geometry may have ambiguous effects on Mb, in case of soft interactions. We may take liberty to generalize that crowding agents may destabilize Mb irrespective of the nature of crowder or the shape and size of crowder. However, we can safely conclude that the stability of native state of proteins may be depending on other aspects, in addition, to the excluded volume effects due to crowders.

Moreover, we should put emphasis on the point that ficoll 70 disrupts the structure of Mb by disrupting the heme polypeptide interaction, which in turn might be responsible for the reduction in its stability. In addition, after analyzing the result with respect to pH’s, it was found that the extent of destabilization is pH dependent, i.e., destabilization is greater at pH 7.0 as compared to pH 6.0 and 8.0, in the case of dextran 70. However, in the case of ficoll 70, the maximum destabilization is at pH 8.0.

It had been observed that preferential binding of additives leads to destabilization of the protein [28,68,69]. We were interested to know whether the observed destabilization of Mb (in the presence of ficoll 70 or dextran 70) and structure disruption (due to ficoll 70) were due to protein–crowder interaction. To confirm this statement, ITC measurements were and molecular docking experiments were done. ITC measurements showed that ficoll 70 and dextran 70 interacted with Mb (Figure 5) [46]. Earlier, we have shown that apoMb do not binds to ficoll 70, which confirms that ficoll 70 and dextran 70 are interacting with the heme moiety of Mb [47]. The ITC data was fitted through “oneSite” binding model. The binding parameter values given in Table 3 shows that total change in Δ*H* and Δ*G* is negative hence binding of dextran to the protein is exothermic and spontaneous and the large value of *K*_a_ and smaller *K*_d_ shows binding is strong. The values from the Table 3 shows that binding is greater between dextran 70 and Mb, where *K*a value is greater than the *K*_a_ of ficoll 70 and Mb. This binding model presumes that one or additional ligand molecule binds with equivalent affinity at single site. This result suggests clear interaction between ficoll 70 and Mb within the confines of this experiment.

Earlier, we have compared the effect of dextran 70 and dextran 40 on two different proteins. The study revealed that macromolecular crowding stabilizes α-LA and lysozyme, and dextran 40 is a better protein stabilizer than ficoll 70 and dextran 70. On the mass/volume scale, dextran 40 has more molecules than dextran 70, which results in maximum packing and hence the highest excluded volume.

Earlier work shows that H-bond length between ficoll 70 and heme is 1.8 and 2.3 Å while the H-bond distance of ficoll 70 with serine 92 is 2.8 Å [46]. It is believed that H-bond distance with donor-acceptor in the range of 1.8–2.3 Å as “strong, while distance in the range of 2.5–3.2 Å as “weak or moderate [70]. Ficoll was shown to bind with Mb at average binding energy of −3.9 kcal/mol. Ficoll 70 alters the conformation of Mb and leads to the reduction in its stability. Ficoll interacts with heme via two strong hydrogen bonds and does not interact with polypeptide chain of Mb but with Ser 92 of it [46]. Sankaranarayanan et al. had showed interaction of ficoll 70 with Serine residue of fibrinogen [71]. To seek into the binding mechanism of dextran with Mb, molecular docking studies were performed. The computational studies Figure S2 shows surface view and cartoon structure of the protein with the crowder (dextran dimer) and Figure S3 shows 2D plot of Mb docked with dextran dimer produced by maestro (Schrodinger), which depicts metal coordination, bonding, charge, hydrophobicity, polarity etc. on the protein in presence of the ligand. This figure revealed that dextran dimer interacts with the heme of Mb via 3 H-bonds with bond distances of 2.1, 1.8 and 1.9 Å, Lys 63 with bond distances of 3.0 and 2.7 Å and Lys 45 with bond distances 2.6 and 2.9 Å. Binding free energy released on interaction of Mb and dextran dimer on docking was calculated to be -2.5 kcal/mol, indicates favorable interactions between Mb and dextran dimer.

Zhang et al. was first to report that different crowder molecules have dissimilar effects on human apo α-lactalbumin (apo-HLA) stability and structure i.e., ficoll 70 increased the stability to some extent, dextran 70 significantly increased the stability whereas PEG 2000 led to destabilization of apo-HLA protein. Moreover, they also observed that neither ficoll 70 nor dextran 70 interacts with apo-HLA but weak interaction was observed between PEG 2000 and apo-HLA. It was hypothesized as if the weak non-specific interaction is overcoming the effect of volume exclusion and thus leading to destabilization of apo-HLA protein.

The structure, stability and function of protein are regulated by balance between all the acting forces (either attractive or repulsive), between the solvent and the unfolded or folded state of protein. The hydrophobic side chains exposed towards solvent in case of the unfolded conformation of protein. When the solvent or co-solute molecule interacts favorably to the exposed hydrophobic group then the unfolded molecule will be more stabilized which leads to decrease of the *T*_m_ [72,73].

Understanding of the intracellular environment requires information about how the proteins are affected in the crowded intra-cellular milieu. The effect of crowding [74] arises due to steric repulsions and chemical interactions [75,76]. The crowding effect on the stability of protein mainly focuses on hard core repulsion, which is mainly stabilizing and entropic in nature. Wang et al. investigated this phenomena by calculating the NMR detected amide proton exchange dependence on temperature and utilized these data in order to find out the enthalpic and entropic contribution due to crowding in case of ubiquitin [77]. Unexpectedly, it was observed that the chemical interaction contribution is huge; and in most of the cases it dominates the hard-core repulsion contribution. They have shown that both hard core repulsion and chemical interaction should be considered while investigating the crowding effects. The hardcore repulsion leads to decrease of the available space to proteins. Le Chatelier’s principle led us to conclude that the repulsive force favors the native state since this state is more compacted than that of the denatured state. For the purpose of simplification, hardcore repulsion may be considered as entropic in nature, however, in ‘repulsive potential, both the enthalpic and entropic contributions do exist always. It should be noted that polymer molecules such as dextran and ficoll exhibit conformational change and also solvation with water molecules, suggesting the both contributions of enthalpy and entropy. Geometrical interactions between such polymers and also polymer-protein exhibits the essential role, as has been well established in terms of depletion interaction between different macromolecules.

The Asakura–Oosawa model has been implemented on a large variety of processes, and often presents a good approximation, particularly when large polymeric crowders are involved in stabilizing colloids or proteins [78]. Over the years it was realized, however, that some cosolutes are not excluded purely because of steric interactions [78,79,80]. Specifically, the free energy changes due to added cosolute were associated with a “sticky” or “soft” attraction component that mitigates the Asakura–Oosawa steric stabilization [78,79]. This additional attractive component is most probably the result of the complex interactions between cosolute, macromolecule, and water. It has been observed that the net emerging depletion force is entropically dominated (in the spirit of the Asakura–Oosawa model) but is counteracted to some extent by unfavorable enthalpy, due to cosolute attraction to the macromolecule [80].

The original concept of macromolecular crowding [81] and massive work in this area has provided importance only to hardcore repulsions however, Interactions can be attractive or repulsive. The repulsive interaction stabilizes the protein because it strengthens the hardcore repulsion. The attractive interaction destabilizes the proteins, e.g., urea interacts with protein leading to their destabilization. Non-specific and favorable interaction with the backbone of protein leads to surface exposure leading to its unfolding. Attractive interactions have enthalpic component [82]. Information regarding the relative effect of chemical interaction and hardcore repulsion is very less because very few studies have shown the effect of crowding on Δ*S*_D_° and Δ*H*_D_°. Crowding stabilizes proteins since the native state is favored due to hardcore repulsion. Crowding increases Δ*G*_D_° because Δ*S*_D_° gets decreased while Δ*H*_D_° remains constant. We can articulate that interactions between macromolecules and ligands are an important part in macromolecular crowding to investigate for the protein stability.

## 5. Conclusions

In this work, the main aim was to know the reason behind the unique behavior of ficoll 70 and dextran 70 towards Mb. It was observed that the degree of destabilization of Mb was greater due to ficoll 70 as compared to that of dextran 70 and was irrespective of the nature of crowder or the shape of crowder (qualitatively independent while quantitatively dependent). The degree of destabilization was observed pH dependent i.e., destabilization is greater at pH 7.0 as compared to pH 6.0 and 8.0, in the case of dextran 70. However, the degree of destabilization was greater at pH 8.0, in the case of ficoll 70. From ITC and computational analysis, it was confirmed that both the crowders (ficoll and dextran) bind to heme moiety of Mb and a single binding site was observed for each. This study advocates that the excluded volume effect is not the only leading factor that determines the conformation of proteins in crowded environment. Soft interactions may also play a major role in the macromolecular crowded environment as far as the stability of a protein is concerned. The central finding of this study is that the destabilization of myoglobin by these crowding molecules is by a weak, but supposedly specific, interaction with the heme.

## Figures and Tables

**Table 1:** Thermodynamic parameters of myoglobin in the absence and presence of different concentrations of ficoll 70 at different pH values.

pH	[Ficoll 70] mg/mL	GdmCl-Induced Denaturation		Urea-Induced Denaturation	
		Δ*G*_D_°(kcal mol^−1^)	*C*_m_[M]	Δ*G*_D_°(kcal mol^−1^)	*C*_m_[M]
6	0	10.13 ± 0.11 (10.15 ± 0.12) *	1.26 ± 0.04 (1.25 ± 0.05) *	10.25 ± 0.18 (10.23±0.15) *	3.99 ± 0.05 (4.00 ± 0.04) *
	100	9.70 ± 0.18	1.15 ± 0.06	9.75 ± 0.15	3.60 ± 0.03
	200	9.22 ± 0.12	1.00 ± 0.05	9.31 ± 0.11	3.30 ± 0.04
	300	8.78 ± 0.13	0.95 ± 0.05	8.72 ± 0.10	3.07 ± 0.06
7	0	11.10 ± 0.14 (11.13 ± 0.16) *	1.53 ± 0.03 (1.56 ± 0.02) *	10.95 ± 0.15 (10.98 ± 0.13) *	5.10 ± 0.04 (5.09 ± 0.05) *
	100	10.34 ± 0.15	1.4 ± 0.04	10.38 ± 0.16	4.63 ± 0.05
	200	9.87 ± 0.11	1.2 ± 0.05	9.87 ± 0.14	4.15 ± 0.06
	300	9.08 ± 0.14	1.1 ± 0.03	9.11 ± 0.13	3.58 ± 0.04
8	0	10.23 ± 0.15 (10.21 ± 0.13) *	1.82 ± 0.04 (1.81 ± 0.03) *	10.25 ± 0.11 (10.23 ± 0.14) *	5.83 ± 0.04 (5.85 ± 0.03) *
	100	9.76 ± 0.11	1.7 ± 0.03	9.71 ± 0.18	5.46 ± 0.05
	200	9.28 ± 0.13	1.65 ± 0.04	9.28 ± 0.13	5.04 ± 0.05
	300	8.81 ± 0.14	1.60 ± 0.05	8.81 ± 0.16	4.75 ± 0.06

* Values given in parenthesis are from Δ*ɛ*_409_ measurements. ‘±’ sign with each parameter represents the mean error obtained from the triplicate measurements.

**Table 2:** Thermodynamic parameters of myoglobin in the absence and presence of different concentrations of dextran 70 at different pH values.

pH	[Dextran 70] mg/mL	GdmCl-Induced Denaturation		Urea-Induced Denaturation	
		Δ*G*_D_°(kcal mol^−1^)	*C*_m_[M]	Δ*G*_D_°(kcal mol^−1^)	*C*_m_[M]
6	0	10.13 ± 0.11 (10.15 ± 0.12) *	1.26 ± 0.04 (1.25 ± 0.05) *	10.25 ± 0.18 (10.23±0.15) *	3.99 ± 0.05 (4.00 ± 0.04) *
	100	9.85 ± 0.15 (9.85 ± 0.14) *	1.04 ± 0.02 (1.05 ± 0.03) *	9.88 ± 0.13 (9.83 ± 0.12) *	3.60 ± 0.03 (3.65 ± 0.02) *
	200	9.44 ± 0.14 (9.43 ± 0.13) *	0.98 ± 0.02 (0.98 ± 0.03) *	9.34 ± 0.14 (9.31 ± 0.12) *	3.10 ±0.02 (3.13 ± 0.03) *
	300	9.05 ± 0.13 (9.05 ± 0.14) *	0.85 ± 0.03 (0.85 ± 0.02) *	9.11 ± 0.13 (9.10 ± 0.14) *	3.70 ± 0.03 (3.71 ± 0.02) *
7	0	11.10 ± 0.14 (11.13 ± 0.16) *	1.53 ± 0.03 (1.56 ± 0.02) *	10.95 ± 0.15 (10.98 ± 0.13) *	5.10 ± 0.04 (5.09 ± 0.05) *
	100	10.55 ± 0.13 (10.57 ± 0.16) *	1.47 ± 0.02 (1.46 ± 0.03) *	10.53 ± 0.16 (10.52 ± 0.18) *	4.71 ± 0.03 (4.70 ± 0.03) *
	200	10.11 ± 0.12 (10.10 ± 0.11) *	1.35 ± 0.01 (1.34 ± 0.04) *	10.17 ± 0.14 (10.14 ± 0.13) *	4.34 ± 0.03 (4.35 ± 0.02) *
	300	9.68 ± 0.13 (9.65 ± 0.12) *	1.15 ± 0.03 (1.16 ± 0.02) *	9.72 ± 0.13 (9.69 ± 0.12) *	4.07 ± 0.02 (4.08 ± 0.03) *
8	0	10.23 ± 0.15 (10.21 ± 0.13) *	1.82 ± 0.04 (1.81 ± 0.03) *	10.25 ± 0.11 (10.23 ± 0.14) *	5.83 ± 0.04 (5.85 ± 0.03) *
	100	9.84 ± 0.14 (9.84 ± 0.16) *	1.58 ± 0.03 (1.59 ± 0.02) *	9.85 ± 0.13 (9.84 ± 0.15) *	4.95 ± 0.02 (4.93 ± 0.03) *
	200	9.40 ± 0.11 (9.43 ± 0.13) *	1.36 ± 0.02 (1.35 ± 0.02) *	9.42 ± 0.15 (9.45 ± 0.13) *	4.61 ± 0.02 (4.62 ± 0.03) *
	300	9.03 ± 0.12 (9.01 ± 0.14) *	1.01 ± 0.03 (1.00 ± 0.02) *	9.04 ± 0.17 (9.06 ± 0.14) *	4.46 ± 0.02 (4.44 ± 0.02) *

* Values given in parenthesis are from [*θ*]_222_ measurements. ‘±’ sign with each parameter represents the mean error obtained from the triplicate measurements.

**Table 3:** Thermodynamic parameters for the binding of dextran 70 and ficoll 70 to myoglobin at pH 7.0 and 25 °C.

Crowder	*N*	*K*_a_(M^−1^)	Δ*H*(cal mol^−1^)	Δ*S*(cal K^−1^ mol^−1^)	*K*_d_(µM)	Δ*G*(kcal mol^−1^)
Dextran 70	0.74 ± 0.04	10.6 × 10^5^ (± 0.88 × 10^5^)	−1.95 (± 0.16)	−42.5 (± 0.3)	9.43	−6.83
Ficoll 70	[0.71 (± 0.04)] ^#^	[9.42 × 10^4^ (± 1.08 × 10^4^)] ^#^	[-2.93 × 10^4^ (± 0.50 × 10^4^)] ^#^	[−45.4 (± 0.40)] ^#^	[10.62] ^#^	[−15.77] ^#^

^#^ [47].

**Table 4:** Comparison of ΔΔG_D_° of myoglobin in the presence of 300 mg/mL ficoll 70 and 300 mg/mL dextran 70 at different pH values.

pH	GdmCl-Induced Denaturation		Urea-Induced Denaturation	
	Ficoll 70	Dextran 70	Ficoll 70	Dextran 70
6.0	1.37 ± 0.17	1.1 ± 0.12 (1.08 ± 0.13) *	1.58 ± 0.19	1.12 ± 0.14 (1.15 ± 0.15) *
7.0	1.43 ± 0.20	1.45 ± 0.12 (1.45 ± 0.13) *	1.26 ± 0.19	1.26 ± 0.14 (1.26 ± 0.13) *
8.0	1.6 ± 0.14	1.18 ± 0.12 (1.22 ± 0.14) *	1.62 ± 0.15	1.19 ± 0.16 (1.19 ± 0.13) *

* Values given in parenthesis are from [*θ*]_222_ measurements. ‘±’ sign with each parameter represents the mean error obtained from the triplicate measurements.

## Supplementary Materials

The following are available online at https://www.mdpi.com/2218-273X/10/3/490/s1, Figure S1: The docked complex of myoglobin (heme and its amino acid residues) with dextran 70 (dimer); Figure S2: Two-dimentional plot interaction of myoglobin (heme and its amino acid residues) with dextran 70 (dimer) generated by maestro (Schrodinger).
