# Supplementary material for: Interactions Under Crowding Milieu: Chemical-Induced Denaturation of Myoglobin is Determined by the Extent of Heme Dissociation on Interaction with Crowders
Source: Biomolecules. 2020 Mar 23;10(3):490. doi: 10.3390/biom10030490 (PMC7175338; doi:10.3390/biom10030490)
Supplement: Supplementary file 1 [file biomolecules-10-00490-s001.pdf]

Supplementary Materials

Interactions Under Crowding Milieu: Chemical-Induced Denaturation of Myoglobin is Determined by the Extent of Heme Dissociation on Interaction with Crowders

Khalida Nasreen [1,†], Zahoor Ahmad Parray [1,†], Shahzaib Ahamad [1], Faizan Ahmad [1], Anwar Ahmed [2,3], Salman Freeh Alamery [2], Tajamul Hussain [2], Md. Imtaiyaz Hassan [1] and Asimul Islam [1,*]

[1] Centre for Interdisciplinary Research in Basic Sciences, Jamia Millia Islamia, Jamia Nagar, New Delhi 110025, India

[2] Center of Excellence in Biotechnology Research, College of Science, King Saud University, Riyadh, Saudi Arabia

[3] Protein Research Chair, Department of Biochemistry, College of Science, King Saud University, Riyadh, Saudi Arabia

† Both the authors have contributed equally

* Correspondence: aislam@jmi.ac.in; Tel.: 0091-9312812007

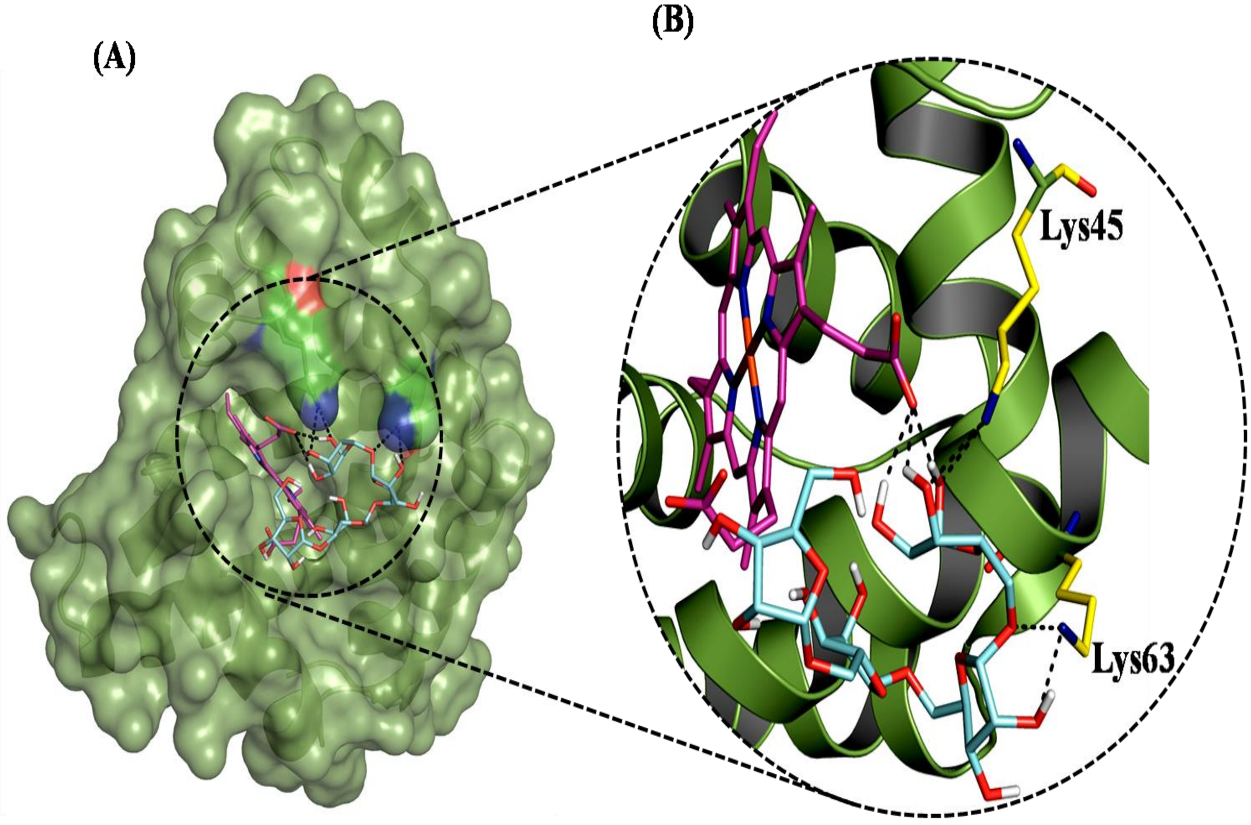

Figure S1. The docked complex of myoglobin (heme and its amino acid residues) with dextran 70 (dimer).

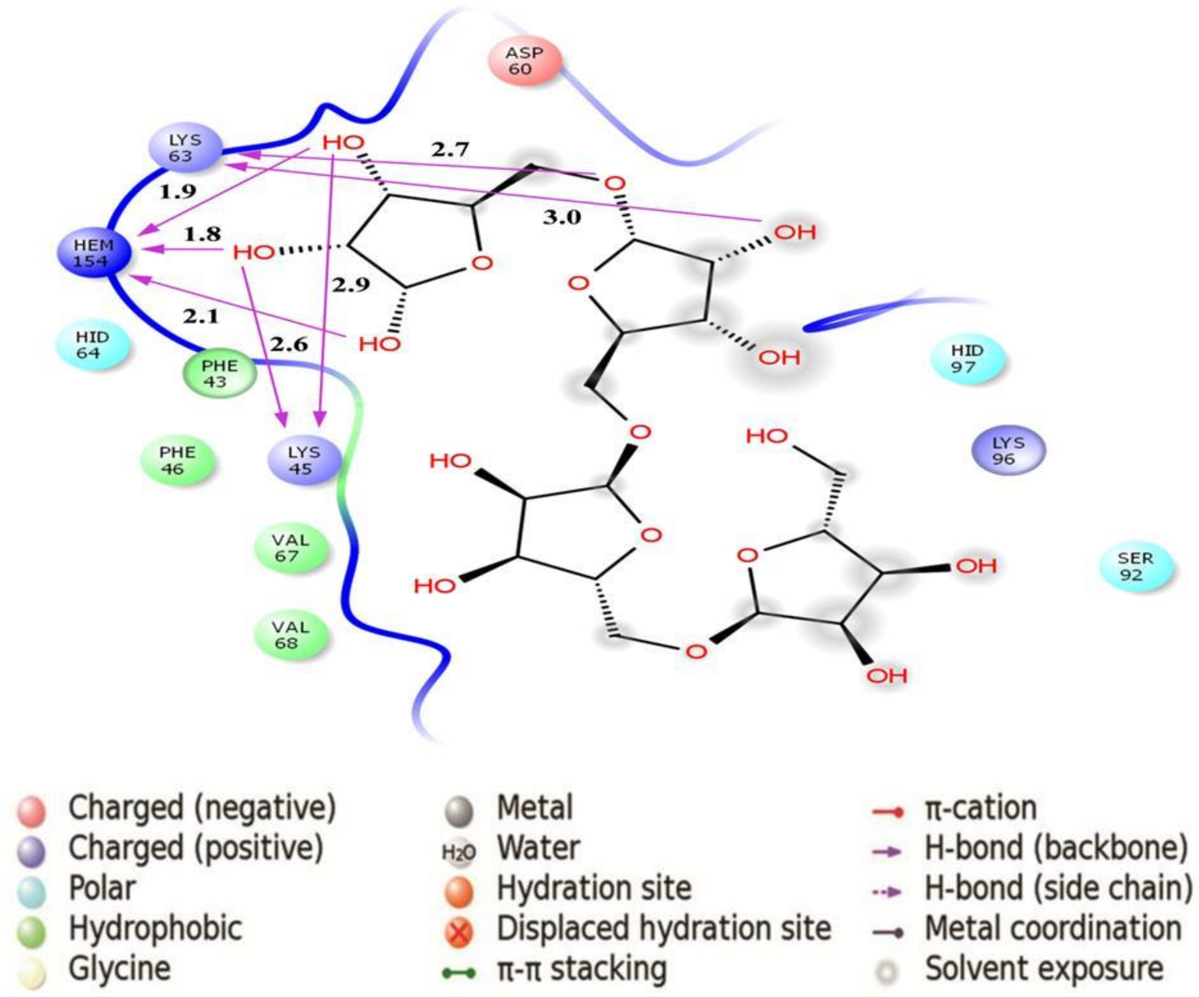

Figure S2. Two-dimentional plot interaction of myoglobin (heme and its amino acid residues) with dextran 70 (dimer) generated by maestro (schrodinger).
